# Delivering Urgent Care Using Telemedicine: Insights from Experienced Clinicians at Academic Medical Centers

**DOI:** 10.1007/s11606-020-06395-9

**Published:** 2021-12-17

**Authors:** Natalie Laub, Anish K. Agarwal, Catherine Shi, Arianna Sjamsu, Krisda Chaiyachati

**Affiliations:** 1 Division of General Pediatrics, The Children’s Hospital of Philadelphia, Philadelphia, PA USA; 2 Center for Pediatric Clinical Effectiveness and Policy Lab, The Children’s Hospital of Philadelphia, Philadelphia, PA USA; 3 Department of Pediatrics, Perelman School of Medicine at the University of Pennsylvania, Philadelphia, PA USA; 4 Penn Medicine Center for Health Care Innovation, Philadelphia, PA USA; 5 Leonard Davis Institute of Health Economics, The University of Pennsylvania, Philadelphia, PA USA; 6 Department of Emergency Medicine, Perelman School of Medicine at the Hospital of the University of Pennsylvania, Philadelphia, PA USA; 7 Department of Medicine, Perelman School of Medicine at the University of Pennsylvania, Philadelphia, PA USA; 8 New York Institute of Technology College of Osteopathic Medicine, Old Westbury, NY USA

**Keywords:** telemedicine, provider experiences, urgent care

## Abstract

**Background:**

Care delivered using telemedicine has been steadily growing in the USA but represented a small fraction of overall visits before the COVID-19 pandemic as few clinicians had been providing care using telemedicine. Understanding how experienced clinicians have practiced telemedicine can help guide today’s exponential adoption of telemedicine.

**Objective:**

The objective of this study was to explore barriers and facilitators to providing effective, high-quality urgent care using telemedicine (“tele-urgent care”) from the perspective of clinicians experienced in telemedicine.

**Approach:**

We conducted semi-structured interviews between July 2018 and March 2019 of clinicians who had been providing tele-urgent care services to patients as a part of their routine clinical practice. Themes were identified using content analysis with a constant comparative coding approach.

**Key Results:**

Among the 20 clinicians interviewed, the majority were female (90%) and nurse practitioners (65%). We identified four themes related to barriers and facilitators to providing effective, high-quality tele-urgent care. Workplace factors such as a strong information technology (IT) infrastructure, real-time IT support, an electronic health record, and a collegial work environment, often virtual, were necessary standards. Communication and exam techniques from in-person encounters were adapted to tele-urgent care including active listening skills and teaching patients to conduct specific exam maneuvers virtually. The convenience of tele-urgent care should be preserved to support improvements in access to care. Finally, patients and clinicians occasionally had mismatched expectations about what could or would be provided during a tele-urgent care encounter. Managing the added tension that can occur during a telemedicine encounter was important.

**Conclusion:**

As telemedicine becomes an integral part of the care continuum, incorporating and accounting for these key insights when we train and support clinicians will be necessary to provide effective, high-quality care to patients in the future.

## INTRODUCTION

Care delivered using telemedicine—virtual visits connecting patients and clinicians through videoconferencing—has been steadily growing in the USA but represented a small fraction of overall visits as few clinicians were historically offering telemedicine to their patients.^1, 2^ In March 2020, the novel coronavirus (COVID-19) pandemic resulted in federal and state de-regulation of telemedicine policies to help facilitate health care’s accelerated shift towards telemedicine to prevent viral spread and maintain access to care for patients where in-person outpatient practices were limiting who could be seen in-person.^3–5^

The monumental transition of the US health care system towards telemedicine has been and will continue being a challenge for clinicians. A national survey of chief executive officers of US health care systems indicated that 38% of health systems had no digital health strategy in 2019.^6^ Even in health systems with digital health strategies, greater investments in technology infrastructure will be required to support clinicians with higher volumes of telemedicine visits, local information technology (IT) expertise to support their ongoing technical needs, and clinicians will need to develop proficiencies and clinical skills when caring for patients using telemedicine. While not all clinicians have historically used telemedicine, some academic medical centers have clinicians who adopted telemedicine early on. Understanding how experienced clinicians have practiced telemedicine can inform how we train and support clinicians who adopt telemedicine into if they are to provide high-quality, safe, and evidence-based care.^7^

The objective of this study was to identify barriers and facilitators to providing effective, high-quality urgent care using telemedicine (“tele-urgent care”) from the perspective of clinicians experienced in telemedicine.

## METHODS

### Study Participants and Setting

We conducted in-depth, semi-structured interviews of clinicians who provided direct-to-consumer, real-time (“synchronous”) telemedicine as a substitute for in-person urgent care appointments at primary care practices or dedicated urgent care clinics (“tele-urgent care”). Tele-urgent care clinicians were purposefully interviewed because tele-urgent care has been commonly employed as a strategy to compete with surrounding urgent care and retail health clinics.^8^ During the study period, few primary or specialty care clinicians had adopted telemedicine due to restrictive payment policies at the state and federal levels.^9^

Participants were recruited from academic medical centers between July 2018 and March 2019 using multiple approaches: direct emails, phone calls, and the email listserv for the American Telemedicine Association’s Academic Medical Center Group. Snowballing techniques were used to identify clinicians who delivered tele-urgent care by asking participants if they knew of other academic medical centers delivering tele-urgent care. Eligible participants included physicians, nurse practitioners, or physician assistants who provided tele-urgent care for either or both adult and pediatric patients. Participants were compensated $50 USD for their time.

### Interview Guide Development

We created a semi-structured, open-ended interview guide (see appendix A) designed to identify barriers and facilitators to providing tele-urgent care. The guide was initially created by authors NL, AKA, and KC. The interview guide was reviewed and pilot tested with two colleagues specializing in qualitative research. It was revised based on this feedback. It was then pilot tested with the first two participants. Pilot testing did not identify any significant content issues, so the guide was not revised any further. Questions were open-ended and included follow-up probes to allow for participants to expand upon answers as needed. Questions included “What do you think are the limitations of providing care via telemedicine?”; “Describe your experience of providing care through telemedicine.”; “What do you imagine are the skills necessary to be a good telemedicine provider?”; and “Describe what you think is different about telemedicine versus in-person care.” Demographic data, including age, gender, race, specialty, occupation, and years in clinical practice were collected from study participants at the completion of each interview.

### Data Collection

Semi-structured interviews were conducted in person or over the phone by three study team members (NS, CS, AS). Interviewers received training from an experienced qualitative methods researcher (NS). Training included meetings to develop and refine the interview guide, direct observation of two interviews, and feedback to enhance the quality of interviews. Interviews lasted approximately 30 min.

### Data Analysis

Interviews were recorded, professionally transcribed, and subsequently coded and analyzed in NVivo 12.0 (QSR International). The interview content was analyzed using the constant comparative coding approach.^10–12^ We began our analysis with codes aligned to the interview guide. During the coding process, we labeled sections of transcripts with the established codes. Three study team members (NS, CS, AS) coded the first four transcripts and developed consensus definitions for codes. Two more transcripts were coded by the study team (NS, CS, and AS), resulting in 30% of the interviews being triple-coded. Initial transcripts were re-coded based on emerging themes and final revisions were made to the code definitions. The study team met regularly to examine results and inter-rater reliability, resolving coding disagreements by consensus and clarify code definitions. Records were kept of decisions to ensure consistency in coding. The average inter-rater reliability was 0.74 with a range of 0.59–1.00, noting excellent reproducibility.^13^ Sections of the interview transcripts representing the study’s main objectives were reviewed to discover additional emerging themes and identify representative quotations. All study protocols and instruments were reviewed and deemed exempt by the University of Pennsylvania’s Institutional Review Board.

## RESULTS

A total of 20 clinicians from academic medical centers in the Northeast, Midwest, and South were interviewed prior to thematic saturation. Ninety percent were female, 61% were White, 90% practiced in the Northeastern portion of the USA, 50% specialized in family medicine, 65% were nurse practitioners, 30% were physicians, 5% were physician assistants, and the average number of years (SD) in clinical practice was 9.7 (9.2) (see Table 1). Four major themes were identified and are listed below (see Table 2).

**Table 1:** Characteristics of Population Interviewed

Characteristic	Study group (*n* = 20)
Age, mean (SD)	43.9 (8.9)
Gender, no. (%)	
Male	2 (10)
Female	17 (90)
Race, no. (%)	
White	11 (61)
African American	5 (28)
Asian	2 (11)
Declined to respond	2 (11)
Region of practice, no. (%)	
Midwest	1 (5)
Northeast	18 (90)
South	1 (5)
Specialty, no. (%)	
Family medicine	10 (50)
medicine	5 (25)
pediatrics	5 (25)
Occupation, no. (%)	
Nurse practitioner	13 (65)
Physician	6 (30)
Physician assistant	1 (5)
Years in clinical practice, mean (SD)	9.7 (9.2)

**Table 2:** Themes and Illustrative Quotes

Themes	Illustrative quote
Workplace factors can promote the delivery of high-quality care	
Reliable videoconference technology infrastructure with real-time information technology support	“I expect to have good quality technical connections, quality video, quality sound” “You have to have champions that are willing to put up the costs as far as equipment and enough technical support when there are issues. The best way to create lack of buy in is when your equipment fails”
Easy access to electronic health records	“I do a pre-chart check so that when I am in my visit I can quickly get to where I have to get and confirm everything”
Supportive clinical team environment, even if virtual	“We have a lot of support with our group. If I ever have questions about anything or concerns all I have to do is text one of my collaborative or my director.”
Clinicians adapted communication and exam techniques from in-person care to tele-urgent care encounters	
Prior experience with in-person care	“In order to do Telemedicine, you need some level of experience. You have to be very comfortable either diagnosing or recommending a patient to do certain things” “My skills have to be sharp enough by just looking at the patient and observing, and hearing the history, to determine if they are sick or not sick.”
Effective use of non-verbal cues	“All of the cues the patient just gave me are the same as if I am in the room with them.” “I thought maybe I would lose clues on the video…I found it interesting that all the cues that I would have picked up on if I was physically with the person, they’re really still there”
Practicing intentional and active listening	“Interviewing is the biggest thing for telemedicine because you don’t have the full exam. You have to really learn how to be a good interviewer” Be a good listener, make sure you are looking at the patient, not typing. Be open and approachable and don’t cut them off” “Give the patient time to talk and don’t put words in their mouth. I had to learning that because I am a talker”
Medical exam	“Try to be very specific, even if it takes a little longer. Even use your body as a model. Demonstrate for them” “You can have someone lay down and assess their abdomen” “I’ll say ‘I want you to listen to what I say then do it’ I want you to sit up straight take a deep breath and blow out. When then do that it allows me to see…can they get a full breath, did they cough, did they wheeze, can they immediately speak?”
Confidence	“You’re trying to get somebody to be confident in what you are deciding over the computer. If you are awkward then maybe the patient would be unsure and not feel good about it”
Patient education	“You need a lot of patient education, its more talking than really examining so a lot of patient education is needed in telemedicine” “Patient education is a big part of it. I think sometimes patient education is forgotten.”
The convenience of telemedicine was beneficial to patients	
Improved access to care	“If there is difficulty getting somewhere, if they are rural, or they can’t drive it give them access to care without some of those other worries”
Potential to avoid the inconvenience or cost of in-person visits	“We don’t have to be face to face to diagnose and treat a lot of conditions” “They don’t have to leave in the middle of the night and waited in an overcrowded ER” “Parents don’t have to take off work, pick their child up from school, call the doctor, see if they can get an appointment, or go to an ER”
Mismatched expectations between patients and clinicians resulted in added tension during the clinical encounter	
Patient expectations for convenient prescriptions	“You need to make sure you know what you are treating. I remember I had a dad that googled symptoms and said my ‘son has an ear infection; can’t you just call in antibiotics.’ I advised his son to come in for a visit and his son actually had pneumonia”
Patient with complex needs looking for quick fixes	“Some people come with a lot of issues and with telemedicine you can’t always provide that level of care.”
Bridging the gap between mismatched expectations	“I will say, ‘hey can you just do me a favor, it will make me feel better. I am sorry if I have taken up your time, but I just want to take really good care of you, and you need to get looked at in person”

### Theme 1: Workplace Factors Can Promote the Delivery of High-Quality Care

Workplace factors consisted of system-level factors that were essential for providing high-quality care, including (a) reliable videoconference technology with real-time IT support; and (b) easy access to the electronic health record (EHR) to review patients’ medical history and communicate with other clinicians, either when consulting or to hand off patients’ care needs. The need for reliable videoconference technology with real-time IT support was described in the following ways,

I’ve only had one frustrating experience because of the video. The patient couldn’t see me…we didn’t know how to fix the problem.

There needs to be an [IT] person ingrained into the team because, and an IT person that understands all the intricacies of the connection. When our IT guy was on vacation, the other people had no idea, they didn’t understand all the pieces

The need for easy access to the electronic health record was highlighted here,

Truthfully [tele-urgent care] the easiest visit in the world, in an office visit there are so many buttons to click and 12,000 things you have to do before you can sign a chart. In tele-urgent care charting is so easy, you don’t need 25 minutes, you report the complaint, the diagnosis and the plan, then sign the chart.

Most clinicians highlighted the need to feel connected. This was described in two ways. First, clinicians believed a supportive clinical team environment was necessary, even if other colleagues or administrators were remote or virtual. Clinicians relied on other colleagues to review cases and felt more confident in their medical decision-making when they could discuss cases with others. Similarly, clinicians wanted to feel connected to the larger health care system. Second, even in a tele-urgent care model, where continuity of care may be disrupted with individual primary care clinicians, clinicians felt patients needed to have continuity of care within the larger care network. The EHR was used to electronically communicate patient visits and clinical decision-making to patients’ primary care provider or specialists, if they were using the same EHR.

### Theme 2: Clinicians Adapted Communication and Exam Techniques from In-Person Care to Tele-urgent Care Encounters

The need for developing virtual competency by using lessons from in-person care was frequently discussed. Clinicians suggested that core competencies in virtual care were important and essentially learned skills, as none received any formal training prior to providing care through telemedicine. To develop these skills, many clinicians reported the importance of having prior in-person clinical experience before practicing tele-urgent care, such as,

In order to do telemedicine, you have to have experience. You have to feel very comfortable diagnosing or doing certain things such as going to the emergency room. Not having experience could make it very daunting.

In telemedicine my skills have to be sharp enough that by just looking at the patient, observing, hearing the history I can determine if they are sick, not sick, if they need to be treated right now or if they can try things at home.

Giving the patients time to express their concerns while displaying intentional and active listening was also identified as essential to achieve a high-quality virtual visit. Clinicians were more attune to these needs during a telemedicine encounter, as highlighted by the following,

Be a good listener, give the patient time to talk. Don’t put words in their mouth. I had to learn this because I am a talker. I would be like ‘is the pain dull’ and I would be putting words in their mouth and that’s really not what you want to do.

Based on prior in-person care experiences, clinicians believed the essential skills needed to provide effective virtual care included non-verbal cues (e.g., body language) and the creative capacity to help aid patients in examination techniques.

I use my body as a model. I demonstrate (lifting arm) because I can’t do it for them. I show them on my body and ask, ‘okay you have a pain here does it go down to here.’

I had a woman palpate her own abdomen…I was like ‘I’m really sorry to tell you this but you likely have acute appendicitis and need to go to the ED.’ She was in the OR within 2 hours.

In addition, clinicians identified the need to exhibit confidence when they had made a clinical decision because patients might be questioning their diagnosis or escalation plan because of the virtual platform. Finally, because time spent examining patients was reduced in the virtual environment, time could be substituted by longer, more in-depth education and counseling relative to what the clinician would have provided during an in-person encounter.

### Theme 3: the Convenience of Tele-urgent Care Was Beneficial to Patients

From the clinicians’ perspectives, the convenience aspect of tele-urgent care made it an important delivery option because patients benefited from better access to care. Patients could avoid the time inconvenience of seeking an in-person visit as described here,

In our school telemedicine program, parents don’t have to take off work, we are able to see them and treat them so that is a huge benefit for our patients.

Patients gain convenience, they don’t have to leave their home, maybe they’re calling at 2 am, and maybe transportation is an issue, maybe cost.

The ability to provide timely reassurance to a patient or family was an important aspect of quality which tele-urgent care facilitated. Clinicians provided a number of examples where patients were at home worried, such as,

Number one they gain some reassurance… [Parents] are really concerned about their child and they want someone just to look at them. So, I think that’s truly the best part of telemedicine.

When conditions could be managed virtually, the convenience of tele-urgent care had the potential to reduce patient’s health care costs (e.g., co-pays for some in-person visits).

Sick visits are sometimes just so silly, when I have to see them in the office. I’m like ‘yea you have poison ivy.’ That you (the patient) have to come in, pay a co-pay and have me look at it to confirm, seems silly sometimes. Telemedicine would eliminate a lot of that.

### Theme 4: Mismatched Expectations between Patients and Clinicians Resulted in Added Tension during the Clinical Encounter

Clinicians perceived added tension during some telemedicine encounters because of mismatched expectations between them and patients. Clinicians believed that the convenience of using telemedicine carried over to patients’ expectations to receive prescriptions or diagnostic testing more easily, as expressed here,

“I think sometimes with the virtual visit it feels kind of consumeristic, meaning I paid for this visit, I expect to get something out of it. Meaning a prescription.

Before I contact the patient, I’m thinking to myself, ‘Oh god, what if I don’t think they need an antibiotic, and this becomes an argument?’

Clinicians highlighted the perception that patients were looking for quick fixes and instant gratification.

It’s a world of instant gratification now, with everything at our fingertips. I think people are pleased that telemedicine is available, and we feel it as clinicians. For me I can hear the satisfaction in their voice. We live in a busy world where everyone is multi-tasking.

The consumerism experienced by tele-urgent care clinicians resulted in many having to develop routinely used skills when navigating the tension between providing evidence-based care, while not wanting to disappoint patients. Many clinicians began to routinely emphasize to patients that they wanted to do what was best for the health of the patient, even if that meant they were not going to meet the patient’s expectations.

## DISCUSSION

As health systems integrate telemedicine into how they deliver care today and beyond, supporting and training the current workforce to be skilled at telemedicine while achieving quality, safe, and evidence-based care will be important. Perspectives and lessons from more experienced telemedicine clinicians can help guide how health systems, educators, and medical societies approach this challenge. In our qualitative study of experienced clinicians, we identified four key facilitators and barriers to delivering tele-urgent care.

First, workplace factors such as a strong information technology (IT) infrastructure, real-time IT support, an electronic health record, and a collegial work environment are necessary standards. Health systems and administrators need to adequately invest in infrastructure (e.g., high-speed broadband and hardware) to support the technical requirements for a high-quality videoconference encounter. While our study is from the perspective of the clinicians, we recognize that telemedicine is a dyad interaction between clinicians and patients. Even if the clinicians have access to the right technology, patients may not. Closing this technology gap will be important especially as we try to reduce disparities in care experienced by low-income and minority patients. Real-time IT support was frequently emphasized. Many of the clinicians interviewed staffed tele-urgent care after hours. Having accessible IT support for virtual support beyond regular business hours will important to maintain patient satisfaction and ensure effective clinical care.

Administrators and managers need to create a team-like atmosphere and foster connections between colleagues even in a virtual practice. Clinicians commented that clinical and collegial support were necessary for effective patient care and was tied to their satisfaction as a tele-urgent care clinician. At a broader systems level, tele-urgent care programs should be integrated within the greater health care system or care network to simplify referrals and streamline communications between tele-urgent care clinicians and primary care providers or specialists. The EHR facilitated these communications.

Second, communication and exam techniques can be adapted to tele-urgent care from in-person encounters. Active listening skills were used by all clinicians when caring for patients. While active listening is vital for fostering a therapeutic relationship during in-person encounters, patients may have a higher awareness of the clinician’s body language during telemedicine encounters. Most videoconferencing technology only allows the patient to visualize the clinicians head to their upper torso. Therefore, patients may be increasingly critical if clinicians look away from the camera, even if the clinician is reviewing the patient’s medical record on an adjacent screen. The videoconference setup limits the patient’s ability to contextualize what the clinician is doing when not maintaining eye contact with the camera. Changing clinicians’ body language or eye movements will require frequent feedback from patients, simulated patient experiences, and self-awareness. Facilitating these changes is seemingly basic but will likely be important for generating positive patient experiences.

Clinicians commonly had patients replicate exam maneuvers that would guide decision-making, including abdominal examinations and active range of motion maneuvers for joints. While clinicians universally recognized that exam options were limited and vital signs were more difficult to collect, clinicians strived to gather as much information as possible to guide the patient’s next steps such as watch and waiting or proceeding to the emergency department. Training clinicians to be comfortable with what is possible and impractical during telemedicine encounters will be important if telemedicine is to be used effectively.

Third, clinicians in our study overwhelmingly felt that the convenience of tele-urgent care was beneficial to patients and should be maintained. Telemedicine provided access to care that patients expressed having struggled with in the past. Patients could avoid time spent traveling or higher co-pays in emergency department settings when conditions could be managed using tele-urgent care. At the same time, clinicians recognized tradeoffs that occurred when providing a more convenient form of care. Patients may seek advice for conditions they might not have seen a provider for in the first place. For the clinicians we interviewed, the benefits of convenience outweighed the costs of overuse. Should health systems and payers create more barriers to care, the gains in access that patients achieve through tele-urgent care may be lost and preserving access to care was felt to be an important principle to uphold.

Fourth, telemedicine visits were occasionally perceived as confrontational. Clinicians were acutely aware of “pressure” from patients to prescribe medications such as antibiotics, and clinicians worried about dissatisfying patients. The risk of dissatisfaction was felt highest among patients that were technologically savvy and were more likely to prefer more convenient care options. The experience of these clinicians may reflect a higher proportion of telemedicine users preferring an informed or consumerism style of clinical decision-making, as opposed to patients who prefer shared decision-making or paternalism.^14^ Patients who prefer consumerism can be as high as 28% of patients and is characterized as patients who do not often deliberate treatment options with clinicians and independently choose which treatment plans should be implemented.^15^ Patients using tele-urgent care may be more likely to represent patients who prefer a consumerism approach to clinical decision-making.

At the same time, clinicians want patients to be satisfied with their encounter and face pressures to practice evidence-based medicine. The countervailing forces of patient preferences and clinicians’ desires to achieve high patient satisfaction may partly explain the high rates of inappropriate antibiotics prescribed among tele-urgent care clinicians.^16–18^ Health care systems and clinicians should consider structured, guideline-driven approaches to support clinical decision-making within tele-urgent care practices. Effective examples include evidence-based contraceptive prescribing practices using direct-to-consumer telemedicine approaches.^19^ Behavioral economic principles could be tested through nudges and default options when managing common conditions encountered in tele-urgent care with the goals of reducing overprescribing of clinically inappropriate medications and unnecessary testing. While understanding how to implement, monitor, and enforce evidence-based practice is universally important for in-person and virtual encounters, tele-urgent care clinicians may face unique pressures if patients are more likely to prefer a consumerism style of decision-making. Therefore, clinicians will benefit from greater training and practice working with patients who prefer a consumerism approach to care.

Limitations to our study include selection bias. Clinicians who enjoy using telemedicine were most likely to agree to participate and early adopters of telemedicine are likely to be positive about their early telemedicine experiences. The participants interviewed here likely represent early adopters within academic medical centers and thus may have represent a cohort of clinicians who fundamentally support health care’s shift towards virtual care and have greater interests in seeing virtual care expand. Therefore, they may be less critical of virtual care. Late-adopters or non-adopters were not interviewed in this study. The sample of clinicians was limited to employees at large academic medical centers prior to the COVID-19 pandemic, limiting the generalizability of our findings to today’s clinicians. Additionally, these larger centers may have more experience in deploying telemedicine and resources to support staff and IT which may bias the sample. While the sample size may be perceived as small, extensive efforts to identify additional clinicians for interviews were employed and thematic saturation was reached. Despite these limitations, we feel that the perspectives of a diverse set of clinicians providing tele-urgent care is an important contribution to the limited literature and provide important, universal insights for clinicians who are learning how to deliver high-quality telemedical care.

## CONCLUSION

Clinicians with experience providing tele-urgent care identified key areas which promote quality care including workplace factors such as a strong technology infrastructure, adapting communication and exam techniques from in-person encounters, preserving tele-urgent care’s convenience, and managing to adhere to evidence-based treatments despite potential mismatched expectations from patients. Health systems, educators, and medical societies should use these insights to guide how they educate, train, and equip clinicians to provide quality, safe, and evidence-based care using telemedicine. Understanding how best to deliver care using telemedicine will serve today’s clinicians and guide health care’s future.
